# Dynamic response to short-pulsed U-ion beams of material candidates for vacuum beam windows manufacturing

**DOI:** 10.1016/j.heliyon.2024.e40707

**Published:** 2024-11-28

**Authors:** Lorenzo Notari, Michele Pasquali, Federico Carra, Marcello Losasso, Jorge Guardia-Valenzuela, Marilena Tomut

**Affiliations:** a DISAT, Department of Applied Science and Technology, Politecnico di Torino, Corso Duca degli Abruzzi 24, Torino, 10129, Italy; b Sapienza University of Rome, Via Eudossiana 18, Rome, 00184, Italy; c Conseil Européen pour la Recherche Nucléaire, Esplanade des Particules 1, Geneva, 1211, Switzerland; d University of Münster, Schlossplatz 2, Münster, 48149, Germany; e GSI Helmholtzzentrum für Schwerionenforschung, Planckstrasse 1, Darmstadt, 64291, Germany

**Keywords:** Dynamic material response, Vacuum beam windows, Thermo-mechanical behaviour, Particle beam impacts, Quasi-instantaneous heat deposition, Stainless steels, Nickel-based superalloys, Aluminium alloys, Titanium alloys

## Abstract

The introduction of next-generation extremely energetic particle accelerator facilities, such as the High-Luminosity upgrade of the LHC (HL-LHC) or the proposed future circular collider (FCC), will dramatically increase the energy stored in the circulating particle beams. This will critically affect the thermo-physical and mechanical properties of the materials adopted, possibly compromising their reliability during the operating lifetime. In this scenario, it is paramount to assess the dynamic thermo-mechanical response of materials presently used, or being developed for future use, in beam intercepting devices exposed to potentially destructive events caused by the impact of energetic particle beams. The present work illustrates the results of an extensive experimental campaign aimed at investigating the thermo-mechanical performances of various materials selected for vacuum beam window manufacturing. The experimental tests described in this study were carried out to explore different phenomena concerning the impact of ion beams on material targets, such as heat deposition and propagation, dynamic response of the samples, and change of mechanical properties as the dose accumulates. The obtained results, evaluated against preliminary numerical investigations and post-irradiation examinations, confirmed the choice of the selected materials as potential candidates for the manufacturing of vacuum beam windows.

## 1 Introduction

The increasing power of next-generation particle accelerator facilities, such as the High-Luminosity upgrade of the LHC (HL-LHC) and the Facility for Antiproton and Ion Research (FAIR), requires the use of materials capable of withstanding extreme irradiation conditions [1], [2]. The interaction of high-intensity particle beams with the components inside the accelerator beamlines and, in particular, with the beam-intercepting devices — targets, beam windows, beam dumps, collimators — may affect the thermo-physical and mechanical properties of the materials adopted and compromise their reliability during the operating lifetime [3], [4], [5].

Numerous experimental campaigns of the past decade have been focused on the characterisation of candidate materials for critical accelerator components under high-energy irradiation conditions [6], [7], [8]. These experiments, which are being carried out at prestigious facilities for material testing, such as the Brookhaven Linac Isotope Producer at BNL [9], [10], the HiRadMat facility at CERN [11], [12], the M-branch experimental beamlines at Darmstadt GSI [13], are essential to verify the suitability of the selected materials for applications in accelerator beamlines.

Building on this foundational research, this study addresses these gaps by providing novel experimental data on the impact of ion beams on material targets. The work presented in this article illustrates the results of irradiation experiments, held at the GSI Helmholtz Center for Heavy Ion Research in Darmstadt (Germany), aimed at investigating beam-induced property changes of certain materials considered appropriate for vacuum beam window manufacturing. This component, essential within particle accelerator complexes and accelerator-based neutron source facilities, consists of a thin interface of separation, located between a volume under vacuum (beam vacuum line) and a volume at higher pressure, and traversed by particle beams. The window must ensure the passage of particles with minimal distortion of the beam and minimum heat deposition, therefore low-Z materials and very small thicknesses are desirable. On the other hand, this thin interface must be able to withstand the required differential pressure between the two environments and resist the enormous thermomechanical stresses induced by the particle beams passing through. The materials from which a beam window is made must be capable of enabling proper and safe operation of this component in the accelerator complex throughout its lifetime. Among the essential requirements, it is important to include high transparency to high-energy particles (low density and atomic number), excellent thermal conductivity to efficiently dissipate the heat deposited by the particle beam inside the target material, high mechanical robustness to withstand pressure difference and beam-induced thermal repeated loads, impermeability to gasses to guarantee absolute leak tightness and resistance to radiation and corrosion. In addition, there are other more general, but not less important, aspects to be taken into account, such as material availability, manufacturing feasibility, production costs, etc.

Each beam window is designed to meet the specific needs of the environment in which it is installed. For example, beam windows for the transmission of low-energy electrons and photons, as in the case of X-ray transmission windows, require very light materials, so that low-Z materials such as beryllium and, more recently, graphene and other membrane materials are indispensable [14]. Excellent particle transmissivity, (minimal heat deposition), high thermal conductivity and exceptional mechanical strength are highly sought-after features of materials for the entrance or exit beam windows of the accelerator facilities' beamlines. Single-layer windows in titanium alloys, austenitic stainless steels and beryllium, and multi-layer structures — where a metallic foil is applied to a carbon-carbon plate to preserve the leak-tightness — are nowadays common in many accelerator facilities (CERN, FNAL, JPARC) [15], [16], [17]. Certain carbon-based materials (Glassy Carbon) and ultra-thin membrane materials (silicon nitride, silicon carbide, graphene, etc.) are actually under investigation to test their feasibility in the next-generation high-energy hadron beam accelerator facilities. In the case of the Spallation Neutron Sources, corrosion resistance, tensile strength and resistance in extremely-irradiated environments become prevalent factors in the selection of the materials that constitute the proton beam windows. Nickel superalloy (Inconel 718) and aluminium alloys are currently the most used materials for such applications. Because of its high corrosion resistance and creep rupture strength, the martensitic stainless steel T91 promises to be a suitable material for beam-window applications in future accelerator-driven subcritical reactors. Table 1 gives an overview of the materials used for particle beam windows, with the corresponding merits and defects, as well as the accelerator facilities where they are actually adopted.

**Table 1:** Materials adopted and investigated for particle beam-window applications in worldwide facilities [18].

Candidate materials	Pros	Cons	Current applications	Envisioned applications
Beryllium (PF-60)	high radiation length, high melting point, high thermal diffusivity	high toxicity, risk of oxidation, manufacturing problems	CERN (HiRadMat [15], CNGS [19]), FNAL [16], [20]	RaDIATE collaboration [7]

Titanium alloys	high specific strength, low coefficient of thermal expansion, high resistance to corrosion and fatigue stresses	low thermal diffusivity	CERN (SPS [21], HL-LHC [22]), FNAL [16], [20], J-PARC [23], [24]	RaDIATE Collaboration, FNAL (LBNF [25], PIP-II [26] to DUNE), ILC

Inconel 718	high tensile strength, high corrosion resistance	low radiation length, low specific heat capacity	LANSCE [27], ISIS (Target Station 1 [28]), SNS (First gen. [29])	

Austenitic steel (AISI 304, AISI 316)	high melting temperature, high corrosion resistance	low radiation length, low specific heat capacity, high coefficient of thermal expansion	CERN (PS, PS Booster, ISOLDE, SPS) [21], [30]	

Martensitic steel (DIN 1.4926, T91)	good thermal conductivity, high melting temperature, excellent corrosion resistance	low radiation length, low specific heat capacity	LANSCE [31], PSI MEGAPIE [32]	CERN irradiation campaigns (IRRAD [33]), JAEA ADS Target Test Facility [34]

Aluminium (Al-5052, AlMg_3_, Al-5083, Al-6061)	high radiation length, high radiation damage resistance, high thermal conductivity, low Young's modulus	low melting temperature, high coefficient of thermal expansion	CERN (SPS [21]), PSI SINQ (Target 3 [35]), ISIS (Target Station 2 [28]), Japan-SNS [17], China-SNS [36], SNS (Second gen. [29])	European Spallation Source [37]

GlassyC (Grade G)	high radiation length, high temperature of fusion, low coefficient of thermal expansion	low thermal conductivity		CERN HiRadMat [38], GSI experiments [39], RaDIATE Collaboration [40]

Carbon Fiber-Reinforced Carbon (Sigrabond-1001-G)	high radiation length, high temperature of fusion, low coefficient of thermal expansion, high thermal diffusivity	high porosity, leak-tightness not ensured	CERN (HiRadMat [15], HL-LHC [22], CNGS [19])	

Our study contributes novel experimental data by exploring different phenomena concerning the impact of ion beams on material targets, such as heat deposition and propagation, dynamic response of the samples, and change of mechanical properties as the dose accumulates. In particular, we present new insights into the thermomechanical response of these materials under such conditions, which have not been comprehensively documented in existing literature, as highlighted in [18]. Cylindrical target samples of the selected materials were exposed to the impact of U-ion pulses, with kinetic energy of 4.8 MeV/u and beam repetition rate of 1 Hz, up to fluence values of $2\cdot 10^{13}$ ions cm^−2^. This detailed analysis not only addresses a significant gap in current research but also validates and enhances the test protocol for future experiments, identifying potential improvements along the way.

The Section 2 contains a brief overview of the materials currently used or planned to be adopted in beam-window applications. Special figures of merit developed for beam-intercepting devices, and introduced to highlight the strengths and weaknesses of each material, assist in the selection process of the target materials which are most worth investigating during the irradiation experiment. The Sec. 3 is dedicated to preliminary thermal and dynamic analyses, performed to predict the behaviour of the sample materials when exposed to the short-pulsed ion irradiation. The Sec. 4 and 5 describe the entire protocol of the irradiation tests, starting from the production of the material specimens and the preparation of the sample holders up to the operational phases of the irradiation experiments. Findings from the in situ measuring instruments — LDV and Thermal camera — are reported in Sec. 6, while Sec. 7 illustrates the results of the two techniques of post-irradiation examinations — microindentation and Scanning Electron Microscopy — used to determine the effect of U-ion irradiation on the microscopical composition and on the mechanical properties of the samples. Finally, the Sec. 8 reports the conclusions of the study, with the most significant outcomes from the measurements and ideas for future developments.

## 2 Selection of materials

### 2.1 Figures of Merits (FoM)

The choice of a particular material for beam windows is driven by the performance of the material under various operational conditions and is based on a number of criteria. In the early stages of design, a set of indices can be particularly useful to orient material choice and to get a clearer picture of the strengths and weaknesses of each material. A common method for classifying and ranking potential materials according to this large number of requirements consists in introducing Figures of Merit (FoM) which allow condensing into a single indicator the multitude of material properties concerning a specific design demand. Two figures of merit deemed important for the design of components subjected to high-energy beam impacts, as the beam windows, are the Thermomechanical robustness index and the Thermal stability index, developed within the Mechanical and Materials Engineering Group at CERN for the design of beam intercepting devices [41], [4].

The Thermomechanical Robustness Index (TRI) is associated with the mechanical robustness of the material and serves to give qualitative information on the material's ability to withstand the impact of a short particle pulse. The following formula can express this figure of merit:

(1) $$\text{TRI}=\frac{R_{\text{M}}\;c_{\text{p}}\;X_{\text{g}}}{\overset{¯}{E}(1-\nu )\;\overset{¯}{\alpha }\;C_{\text{R}}\;\rho^{\text{n}}}\cdot {\left(\frac{T_{\text{melt}}\;c_{\text{p}}\;X_{\text{g}}}{C_{\text{R}}\;\rho^{\text{n}}}-1\right)}^{\text{m}}$$

where $\overset{¯}{E}$ [GPa] is the (averaged) Young's modulus, *ν* the Poisson's ratio, $R_{\text{M}}$ [MPa] the failure strength, $T_{\text{melt}}$ [K] the melting (or degradation) temperature, *ρ* [g cm^−3^] the material density, $c_{\text{p}}$ [J g^−1^ K^−1^] the specific heat, $\overset{¯}{\alpha }$ [10^−6^ K] the (averaged) coefficient of thermal expansion, $X_{\text{g}}$ [cm] the geometrical radiation length, *m* and *n* two exponential coefficients and $C_{\text{R}}$ a scaling factor.

The Thermal Stability Index (TSI) indicates a material's capacity of preserving geometrical stability and minimising deformation when subjected to steady-state beam losses. This index can be finally written as:

(2) $$\text{TSI}=\frac{\overset{¯}{\lambda }\;X_{\text{g}}}{\overset{¯}{\alpha }\;C_{\text{S}}\;\rho^{\text{n}}}$$

where $\overset{¯}{\lambda }$ [W m^−1^ K^−1^] is the (averaged) thermal conductivity and C_S_ represents a scaling factor. Additional details on TRI and TSI can be found in [18].

### 2.2 Materials selection

**Figure 1:**
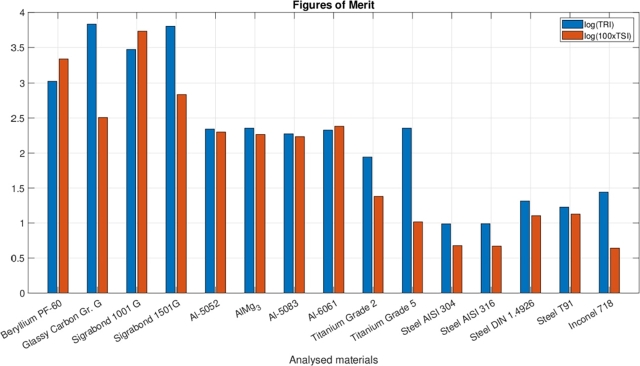
Comparison of the thermomechanical robustness indices (TRI, in blue in the figure) and thermal stability indices (TSI, in red in the figure) estimated for various beam windows materials.

The findings related to the two figures of merit (Eqs. (1) and (2)), originally conceived for beam-intercepting devices, are presented in Fig. 1. The carbon-carbon composites have very high values for both the FoM, which explains why this kind of material has been selected for being used as an absorber for new collimators and why the design of CERN windows of the last decade provided for their use in combination with metallic foils. The only metal with comparable TSI and TRI values to the carbon-carbon composites is Beryllium. Among the other metals, only aluminium alloys have a TSI value that exceeds the unit as well as relatively high values of TRI, very similar to those found for Ti-6Al-4V. The AISI 316 and AISI 304 austenitic steels share very low values of TSI with Inconel 718, but exhibit TRI values around a third lower with respect to the Nickel superalloy and values halved of both the figures of merit with respect to the martensitic steels.

In addition to the considerations emerging from the figures of merit, the selection of materials took into account other aspects, such as availability, non-toxicity and manufacturability. For instance, beryllium was not regarded as an attractive material since, because of its intrinsic toxicity, it was preferred not to incur the risk of its rupture during the shipping phase from Geneva (CERN) to Darmstadt (GSI) or during the irradiation experiment inside the UNILAC M3-Branch beamline.

Out of the several investigated materials, the following downselection was proposed:

• Among the stainless steels, the T91 Steel (9Cr-1Mo) was selected. It exhibits strong corrosion resistance, good creep rupture strength and low coefficient of thermal expansion, and that has been selected for the realisation of the ADS beam windows;
• Inconel 718, a Ni-based superalloy, which has exceptional corrosion resistance and ultimate tensile strength, is particularly interesting in the solution annealing condition for its radiation resistance even under low-temperature irradiation;
• Among the analysed aluminium alloys, generally characterised by excellent thermal conductivity, low density but also high tensile strength, Al-6061-T6 has the best FoM values: this supports its choice as baseline material of the 5W-Proton Beam Window at European Spallation Source (ESS). In fact, given a higher value of tensile strength (other mechanical and physical properties being equal), Al-6082-T6 aluminium alloy was chosen instead of Al-6061-T6;
• Among the titanium alloys, Titanium Grade 5 (Ti-6Al-4V) is characterised by the excellent specific strength and by reduced activation for severe radiation exposure, which is one of the most widely used metals for future beam-window designs in accelerator facilities, such as the HL-LHC at CERN and Long Baseline Neutrino Facility at FNAL. Titanium Grade 5 ELI (Extra Low Interstitials), also known as Titanium Grade 23, was eventually preferred to Ti-6Al-4V because of its reduced oxygen content (0.13%), leading to an enhancement of fracture toughness.

The mechanical and physical properties of the materials selected for the irradiation experiments are presented in Table 2. The data reported in the table were obtained from technical data sheets of the various manufacturers or, if unavailable, from online reference data sheets for the specific material [42], [43], [44], [45].

**Table 2:** Physical and mechanical properties of the candidate materials selected for GSI irradiation tests.

		Steel	Inconel	Al	Ti
		T91	718	6082	Gr. 23
Physical properties					
Density	*ρ* [g cm^−3^]	7.77	8.23	2.70	4.43
Thermal conductivity	$\overset{¯}{\lambda }$ [W m^−1^ K^−1^]	27.00	11.40	180.00	6.70
Specific heat capacity	c_p_ [J g^−1^ K^−1^]	0.46	0.44	0.90	0.53
Thermal diffusivity	a [mm^2^ s^−1^]	7.55	3.18	74.07	2.87
Melting temperature	T_melt_ [^∘^C]	1450.00	1298.00	650.00	1630.00
Volumetric CTE	$\overset{¯}{\alpha }$ [10^−6^ K^−1^]	11.30	13.00	23.60	8.60

Mechanical properties					
Young's modulus	E [GPa]	207.00	203.00	70.00	113.80
Poisson's ratio	*ν*	0.30	0.29	0.33	0.34
Yield strength	YS [MPa]	415.00	1100.00	255.00	790.00
Ultimate tensile strength	UTS [MPa]	585.00	1375.00	300.00	860.00

## 3 Preliminary thermo-mechanical analyses

**Figure 2:**
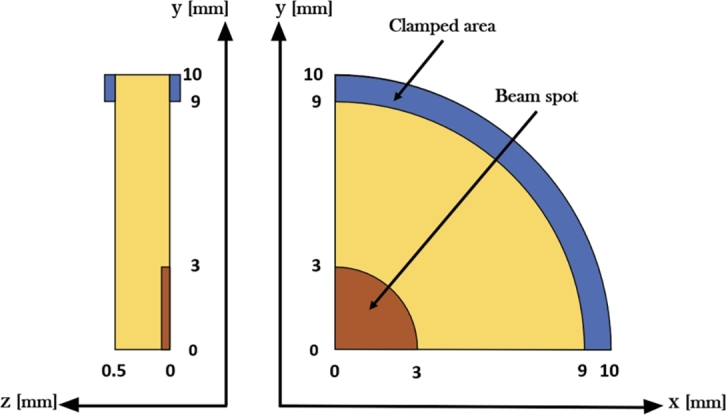
Schematic drawing in side and front views of a quarter of the target disc, where *z* is the direction of thickness and *x* and *y* are the directions of the plane of the disc. All units are in mm.

Preliminary analytical calculations were performed with the aim of predicting the thermal and dynamic behaviour of the material samples when irradiated with U-ion beams, in preparation of the experimental campaign at GSI. The Uranium ions are expected to hit the disc-shaped target samples (with a diameter of 20 mm and thickness lower than 0.5 mm) in the centre, as can be seen in the Fig. 2, resulting in a homogeneous beam spot with a circular shape and with a radius of approximately 3 mm. For ease of readability, the actual geometrical values of the samples after machining were used for the preliminary calculations, in such a manner that the analytical findings are as accurate and close to experimental reality as possible. The samples of the four selected materials will be exposed to 100 μs-long U-ion pulses with a kinetic energy per nucleon of 4.8 MeV/u (1.14 GeV, in total), an intensity of around $5\cdot 10^{9}$ ions/cm⋅2pulse and a beam repetition rate of 1 Hz, in line with the beam parameters of the last U-ion irradiation test held at the M3-beamline of the UNILAC at Darmstadt GSI [13], [39].

### 3.1 Thermal analyses

#### 3.1.1 Estimation of peak and stationary temperatures

The instantaneous temperature rise after a single ion beam impact on the target material can be analytically estimated by the following equation [46]:

(3) $$\Delta T_{peak}=(\frac{dE}{\rho dx})\;\frac{\varphi }{c_{\text{p}}}$$

where dE/ρdx is the mass stopping power, *φ* is the intensity of the U-ion beam, namely the flux of ions passing through the beam spot of the sample per pulse (5⋅10^9^ ions/cm⋅2pulse) and $c_{\text{p}}$ the specific heat capacity. The equation above is valid under several conservative assumptions, which are outlined below:

• The stopping power refers to the energy lost by the passing beam, not necessarily the energy deposited in the target, since a fraction of this energy may escape from the window. This results in a conservative estimation of the energy deposited.
• The ion beam is uniformly distributed across the circular beam spot.
• All the energy of the incident beam is converted into heat due to ionising energy losses.
• The heat does not spread outside the beam spot during the energy deposition, which is reasonable given the expected short deposition time of 100 μs.
• Cooling processes like radiation, which are particularly important at elevated temperatures, have been excluded from this analysis for simplicity.

Furthermore, in this case, as well as in the subsequent preliminary thermo-mechanical calculations, the slight temperature rise during ion beam impact is assumed to have a negligible effect on the material properties, allowing them to remain consistent with their room temperature values. This assumption is validated by previous irradiation tests within the M-branch, where the properties of different metallic alloys under uranium irradiation at the same energy were found to be stable regardless of temperature changes [39], [13]. Therefore, the effects of temperature-dependent properties are considered negligible in these reference analyses.

Since the flux is a constant parameter and the heat capacity is known for all the materials of interest for beam windows, the solution to the equation lies in the research of the values of mass stopping power.

The mass stopping power depends on the amount, the type and the energy of ions impacting the target sample as well as on the density and the physical properties of the material itself. Its calculation can be carried out with extreme accuracy by sophisticated computational algorithms based on Monte Carlo methods, but reliable data for preliminary analyses may be also extracted by nuclear libraries [47], [48].

**Figure 3:**
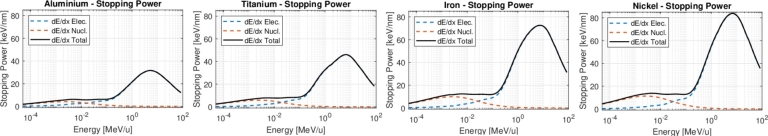
Electronic, nuclear and total energy loss as a function of the specific energy of uranium ions for four elements (Al, Ti, Fe, Ni). All the graphs were realised by processing the data extracted from the SRIM-2013 libraries.

The data relating to stopping power for uranium ions impacting on the constituent elements of the four target materials (Al, Ti, Fe and Ni) were extracted from the SRIM-2013 library and plotted as a function of the kinetic energy per nucleon in the graphs of Fig. 3. The graphs are sorted from the lightest element, Aluminium (Z=13, A=27), for which the Bragg peak is just pronounced, to the heaviest element, Nickel (Z=28, A=58.7), for which the peak is far higher. The total stopping power dE/dx (black curve) is obtained by summing the values of the nuclear stopping power (dashed orange curve) and the electronic stopping power (dashed blue curve).

It is worth noting that the stopping power for 4.8 MeV/u U-ions is almost entirely affected by the phenomenon of electronic loss. At this energy level, uranium ions experience their highest energy losses due to interactions with the target material. This specific energy was chosen to maximise both energy losses and the likelihood of radiation-induced damage in the target specimens. This practice allows for fast dose accumulation in the material samples, as well as efficient use of beamtime during the UNILAC irradiation experiments.

Another important aspect to consider in preliminary analyses is the temperature at the end of the thermal transient provoked by single-pulse irradiation. Assuming that all the heat deposited by the U-ions inside the beam spot has spread perfectly throughout the material sample and has not been dispersed outside, then the temperature rise of the specimen under steady-state conditions can be found directly by the energy conservation equation between the heat deposited in the beam spot $Q_{\text{dep}}$ and the heat absorbed by the material sample $Q_{\text{abs}}$, that causes a rise in sample temperature $\Delta T_{st}$:

(4) $$\left\{ \begin{array}{c} Q_{\text{dep}}=N_{U}\cdot E_{u}\cdot \varphi \cdot (\pi r^{2})\\ Q_{\text{abs}}=\rho \cdot (\pi R^{2}h)\cdot c_{\text{p}}\cdot \Delta T_{st} \end{array}\right.$$

where $E_{u}$ is the kinetic energy per nucleon (4.8 MeV/u), $N_{U}$ the number of nucleons per uranium ion (238), *φ* the number of incident uranium ions on the beam spot per pulse ($5\cdot 10^{9}$ ions/cm^2^ pulse), *r* the radius of the beam spot and *R* and *h* the radius and the thickness of the material sample, respectively. The stationary rise in temperature can be expressed as derived from Eqs. (4):

(5) $$\Delta T_{\text{st}}=\frac{N_{U}\cdot E_{u}\cdot \varphi \cdot (\pi r^{2})}{\rho \cdot (\pi R^{2}h)\cdot c_{\text{p}}}$$

The values of peak and stationary temperatures calculated by the Eqs. (3) and (5), as well the ratio between the peak temperature and the melting temperature, are presented in Table 3. A room temperature of 25 ^∘^C was considered for all the materials. The highest temperatures, around 210 ^∘^C, are expected for Inconel 718 sample, due to large values of mass stopping power and low specific heat capacity, followed in descending order by Titanium grade 23 and steel T91 (around 180-185 ^∘^C), and finally 6082 alloy, with a maximum temperature lower than 130 ^∘^C by virtue of its high value of specific heat capacity. As regards the maximum vs. melting temperature ratio, Titanium grade 23 behaves better than the other metallic materials (${\text{T}}_{\text{max}}$/${\text{T}}_{\text{melt}}<$0.25), while the ratio increases almost up to 0.5 for Al-6082-T6, which have lower melting temperatures than the other materials considered. At the end of the thermal transient, the heat deposited by the ion beam causes a slight increase in temperature of up to a maximum of 5 degrees for the sample in titanium alloy.

**Table 3:** Peak and stationary temperatures estimated for the four materials selected for the UNILAC experiment in the case of a single-pulsed U-ion irradiation. A room temperature of 25 ^∘^C was used for calculations.

		Steel	Inconel	Aluminium	Titanium
		T91	718	6082-T6	Grade 23
Peak temperature	T_peak_ [^∘^C]	185.26	210.33	129.22	181.47

Stationary temperature	T${}_{st}$ [^∘^C]	28.00	27.91	27.32	29.46

Ratio between T_peak_ and T_melt_	T_peak_/T_melt_ [K/K]	0.27	0.31	0.44	0.24

#### 3.1.2 Estimation of heat diffusion times

Another aspect to be considered in the thermal design of a beam window concerns the process of diffusion of heat from the irradiated beam spot at the centre to the edge of the window. Most of the heat produced from the beam impact must be transported away from the centre before the next impact occurs: the heat accumulation at the centre could, pulse per pulse, cause the melting of the window's material and even its failure.

Since the heat propagation rate through a material is properly described by the thermal diffusivity *a*, a characteristic time *τ*, called thermal diffusion time, can be used to estimate the length of the thermal transients:

(6) $$\tau =\frac{L^{2}}{a}$$

where *L* is the characteristic dimension of a physical system. For the thin disc-shaped samples of the irradiation experiment, radius *R* and thickness *h* can be taken as a typical dimension for Eq. (6), from which:

(7) $$\tau_{\text{R}}=\frac{R^{2}}{a}\;\;\;\;\;\;\;\;\;\;\tau_{\text{h}}=\frac{h^{2}}{a}$$

The parameter *τ*, and in particular $\tau_{\text{R}}$ since R≫h, gives an indicative idea of the cooling time scales of the test samples: it is therefore related to the time needed to reach, through heat diffusion processes, a uniform temperature distribution in the region [49]. This relationship is valid under the assumption of quasi-instantaneous heat deposition, namely when the thermal diffusion time is much longer than the duration of beam impact: in the case of the irradiation experiment, in which the length of U-ion beam impact and the beam repetition rate is expected to be 100 μs and 1 Hz, respectively, this condition is definitely met.

The values of thermal diffusion time along thickness and radius are shown in Table 4 for the four materials selected. A strong difference can be observed between Al-alloy (τ=1.35 s) and Ti-alloy, for which the time necessary to establish a uniform temperature distribution on the sample surface is considerably greater. These findings suggest that the peak temperature might significantly increase after several impacts for Titanium alloy, Inconel 718 and steel samples, whereas slight differences in the peak temperature are expected for Al-alloy sample, for which a maximum temperature of 130 ^∘^C is expected to remain stable even after many beam impacts.

**Table 4:** Radial and axial thermal diffusion times for the four cylindrical samples to be investigated under irradiation, as computed from Eqs (7). As a comparison term, it should be recalled that the pulse length is 0.1 ms.

		Steel	Inconel	Aluminium	Titanium
		T91	718	6082-T6	Grade 23
Thermal diffusivity	a [mm^2^ s^−1^]	7.55	3.18	74.07	2.87

Radial thermal diffusion time	*τ*_R_ [s]	13.24	31.42	1.35	34.80

Thermal diffusion time along thickness	*τ*_h_ [ms]	6.03	15.11	2.22	16.83

### 3.2 Dynamic analyses

The pulsed deposition of heavy ions, with energy close to the Bragg peak, induces high thermal stresses on the irradiated targets, from which vibrations, stress waves and other dynamic effects are generated.

#### 3.2.1 Vibration of circular samples

When heavy ions impinge on target samples, their energy is almost entirely converted into heat and rapid growth in temperature occurs quasi-instantaneously inside the beam spot. This rise in temperature causes the thermal expansion of the irradiated material. Since the heating initially affects only one surface of the disc-shaped samples, only the superficial part tends to expand and, as a result, bending modes are generated.

The natural bending frequencies of samples under irradiation can be predicted analytically by treating the samples as circular plates and then by using the fundamental equations of classical plate theory for the precise determination of this characteristic frequency. The solution of the equation of motion for isotropic plate given by the Kirchhoff-Love theory provides a simple formula to compute the bending vibration frequencies for disc-shaped samples [50]:

(8) $$f_{ij}=\frac{\lambda_{ij}^{2}}{2\pi R^{2}}\sqrt{\frac{Eh^{2}}{12\rho (1-\nu^{2})}}$$

where *R* is the “unclamped” radius, *h* is the plate thickness, *ρ* is the mass density, *E* is the Young's modulus, *ν* is the Poisson's ratio and $\lambda_{ij}$ are the roots of the equation, where *i* refers to the number of nodal diameters and *j* to the number of nodal circles, with the boundary circle excluded. The value of $\lambda_{ij}$ depends on the Poisson's number and on the nature of the boundary condition that the target samples will experience under irradiation during the experiment. Since the actual boundary conditions are not known a priori, different values of the eigenvalue $\lambda_{ij}$ for as many boundary conditions that could occur during the experiment were analytically determined.

The boundary conditions which allow for predicting the tightening effect of the rim of the sample holder on the outer radius of the disc-shaped target samples are basically three: the clamped edge, which completely prevents the outer region of the samples to move, the free edge, for which the samples do not perceive the contact with the ring-shaped sample holder, and the simply-supported edge. In addition to these, there is a variety of alternative boundary conditions for which the eigenvalues $\lambda_{ij}$ are included between the simply supported and the clamped condition, such as the case of the elastically supported plates (where springs uniformly distributed about its contour prevent translation and edge rotation) and of the plates with initial tension or compression (in which the effects of in-plane compressions or tensions that may develop in the plate due to thermal or mechanical phenomena are taken into account). The roots $\lambda_{ij}^{2}$ of Eq. (8) for these and other boundary conditions are available in literature [51], [50], [52].

#### 3.2.2 Wave propagation phenomena

The thermal expansion, due to the quasi-instantaneous heat deposition into the beam spot, is initially prevented from spreading by the thermal inertia of the outer material. This results in thermal stresses which propagate in the sample in the form of mechanical waves [53]. The wavefront thus produced propagates from the beam spot throughout the material sample and in all directions. Two types of body waves are generated simultaneously: the P waves (also called primary waves or pressure waves) and the S waves (secondary waves or shear waves). The P-waves are high-velocity tension/compression waves that move from the front to the back surface of the samples and go back, travelling for a distance equal to two times the thickness of the samples (2*h*).

The frequency ($f_{P}$) with which the primary waves move between the front and rear surface of the samples can be calculated as the ratio between the velocity of the P-wave ($c_{P}$) and the distance travelled by the wave before returning to its starting point (2*h*) [54], [55]:

(9) $$f_{P}=\frac{c_{P}}{2h}=\frac{1}{2h}\sqrt{\frac{E(1-\nu )}{\rho (1+\nu )(1-2\nu )}}$$

The shear vertical (SV) waves are a type of elastic wave, caused by the shear stresses inside the beam spot, perpendicular to the direction of wave propagation and with a polarisation perpendicular to the surface plane. The frequency ($f_{S}$) with which this kind of secondary wave moves between the edges of the cylindrical samples can be calculated as the ratio between the velocity of the S-wave ($c_{S}$) and the distance travelled by the wave before returning to its starting point (4*R*, where *R* is the radius):

(10) $$f_{S}=\frac{c_{S}}{4R}=\frac{1}{4R}\sqrt{\frac{E}{2\rho (1+\nu )}}$$

#### 3.2.3 Results of the dynamic analyses

Table 5 presents the natural bending frequencies and the frequencies of propagating waves, determined using the analytical equations for circular plates. The calculations are based on the assumption that all the cylindrical samples have the same size (R=10 mm, h=0.2 mm). The natural frequencies were computed using Eq. (8) for the first two modes of vibration (when i=0): these two modes are expected to be the most excited by the ion beam impact, and thus also those for which making frequency measurements by the Laser Doppler Vibrometer is easier. The values of $\lambda^{2}$ to be included in Eq. (8) were calculated for the boundary conditions of clamped edge, free edge and simply supported edge. The frequencies of the P-waves and of the SV-waves were calculated by Eqs. (9) and (10).

**Table 5:** Analytical results of natural bending frequencies and propagation frequency of waves for the four material samples.

		Steel	Inconel	Al	Ti
		T91	718	6082	Gr. 23
Clamped sample	1${}^{st}$ mode [kHz]	6.69	6.60	12.68	6.88
bending frequency	2${}^{nd}$ mode [kHz]	26.04	25.66	49.32	26.75

Free sample	1${}^{st}$ mode [kHz]	4.78	4.70	9.11	4.96
bending frequency	2${}^{nd}$ mode [kHz]	20.39	20.09	38.68	20.99

Simply Supported sample	1${}^{st}$ mode [kHz]	3.23	3.17	6.17	3.36
bending frequency	2${}^{nd}$ mode [kHz]	19.47	19.18	36.91	20.03

P-wave velocity	*c*_*P*_ [m/s]	5988.55	5683.98	6197.82	6313.39

Freq. of P-wave	*f*_*P*_ [MHz]	14.03	12.96	7.64	14.36

S-wave velocity	*c*_*S*_ [m/s]	3201.02	3091.24	3121.95	3093.70

Freq. of S-wave	*f*_*S*_ [kHz]	80.03	77.28	78.05	77.34

The LDV that will be used during the irradiation experiment has a maximum sampling frequency of 25 MHz. All the frequency values reported in Table 5 fall below this threshold, except for those of the primary waves: it follows that the frequencies corresponding to the P-waves (in the order of 10 MHz) cannot be detected by the instrument in use and cannot be confused with the ones associated with the natural bending modes, whose frequencies record maximum values of tens of kHz. On the contrary, the frequency values of the shear vertical waves are lower than the sampling threshold and are potentially detectable by the LDV.

Table 6 summarises the main results of the preliminary analyses.

**Table 6:** Main results of the preliminary analyses for the four materials selected for the GSI UNILAC irradiation experiment.

		Steel	Inconel	Al	Ti
		T91	718	6082	Gr. 23
Peak temperature	T_peak_ [^∘^C]	185.26	210.33	129.22	181.47

Stationary temperature	T${}_{st}$ [^∘^C]	28.00	27.91	27.32	29.46

Ratio between T_peak_ and T_melt_	$\frac{{\text{T}}_{\text{max}}}{{\text{T}}_{\text{melt}}}$ [K/K]	0.27	0.31	0.44	0.24

Radial thermal diffusion time	*τ*_R_ [ms]	13237.78	31419.21	1350.00	34798.64
Thermal diffusion time					
along thickness	*τ*_h_ [ms]	6.03	15.11	2.22	16.83

Clamped sample	1${}^{st}$ mode [kHz]	6.69	6.60	12.68	6.88
bending frequency	2${}^{nd}$ mode [kHz]	26.04	25.66	49.32	26.75

Free sample	1${}^{st}$ mode [kHz]	4.78	4.70	9.11	4.96
bending frequency	2${}^{nd}$ mode [kHz]	20.39	20.09	38.68	20.99

Simply Supported sample	1${}^{st}$ mode [kHz]	3.23	3.17	6.17	3.36
bending frequency	2${}^{nd}$ mode [kHz]	19.47	19.18	36.91	20.03

Frequency of P-wave	*f*_*P*_ [MHz]	14.03	12.96	7.64	14.36

Frequency of S-wave	*f*_*S*_ [kHz]	80.03	77.28	78.05	77.34

## 4 Production and preparation of the samples for the test

### 4.1 Production of testing samples

The samples of the selected materials were produced in cylindrical shape, with a maximum diameter of 2 cm, so that they could be securely mounted in the ring-shaped sample holders. The thickness of the samples must be minimised as much as possible in a way that the homogeneous irradiation of the beam spot is ensured and that a meaningful correlation between radiation-induced damages (dpa) and material property changes can be produced. The lower limit of this thickness is dictated by the penetration depth of the U-ions inside the matter and, most importantly, by the machinability of the materials, because the cutting techniques do not allow for the fabrication of cylindrical slices from metallic round bars with thicknesses that are lower than the corresponding penetration depths.

The manufacturing of the specimens was executed by cutting round bars into thin cylindrical slices by using electrical discharge machining (EDM). After the cutting, the surfaces of the samples have been polished to take away the oxides created during the cut.

**Figure 4:**
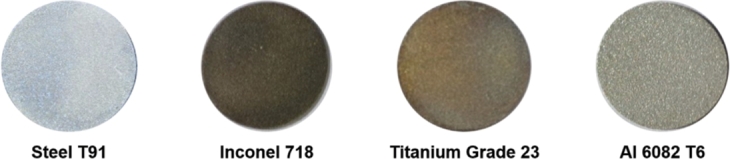
Pictures of T91 steel sample (produced by RHP-Technology) and Al-6082-T6, Titanium Grade 23 and Inconel 718 samples, produced by the CERN Workshop.

The production of the T91 Stainless Steel samples was completely entrusted to the Austrian company RHP-Technology, while the CERN Workshop was responsible for the supply of the other metallic samples. As already mentioned, Titanium Grade 5 ELI (Extra Low Interstitials), also known as Titanium Grade 23, was preferred to the more common Ti-6Al-4V because of its reduced oxygen content (0.13%), leading to an enhancement of fracture toughness. Similarly, Al-6082-T6 was chosen instead of Al-6061-T6 on account of a higher value of tensile strength (other mechanical and physical properties being equal). The IN718 specimens were produced from a 40 mm in diameter round element in precipitation-hardened Inconel 718, which was previously melted, remelted, forged, solution annealed, quenched and double ageing heat treated [43]. The pictures of these samples are reported in Fig. 4.

### 4.2 Samples preparation for the test

**Figure 5:**
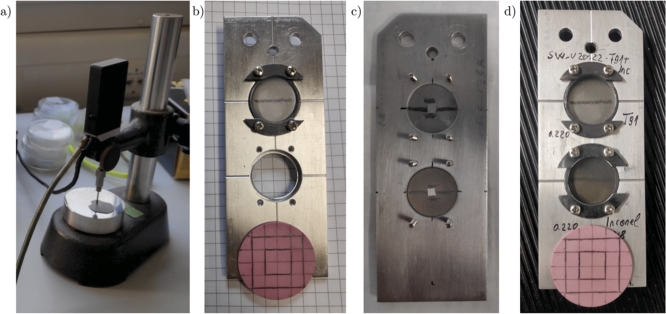
Pictures of the process of sample preparation: (a) high accuracy length gauge to measure the effective thickness of the samples, (b) mounting of the T91 steel sample in the upper housing of the sample holder, (c) back side of the first sample holder, where the application of the graphite spray and of the tiny piece of adhesive reflective tape can be noticed, (d) front side of the first sample holder, ready to be installed inside the spectroscopy chamber.

Once manufactured, the samples were transported to Darmstadt GSI for the irradiation tests. On account of the limited beam time, only one sample for each material was exposed to the uranium-ion beams in the experimental facility. The target samples to be irradiated were chosen according to their effective thickness, measured by using a high accuracy length gauge, as shown in Fig. 5(a).

Different measures of the thickness in various positions of each sample were made and the average and its uncertainty were computed. As regards Steel T91, Inconel 718 and Titanium Grade 23, the samples with the closest average values (around 220 μm) were chosen, while the sample in aluminium alloy with the smallest average value was selected. The preparation procedure of the sample holders can be summarised in the following few steps:

• the samples were mounted in a ring-shaped sample holder, resulting in a free-standing diameter of the disc of 18 mm (Fig. 5(b));
• the four screws were tightened to ensure the best possible contact;
• a conductive graphite spray was used to apply black paintings on the front and on the rear faces of the samples (see the horizontal black lines in Fig. 5) and on the ring surfaces in order to render the samples emissivity as uniform as possible and allow the thermal camera to more accurately acquire temperatures;
• a tiny piece of adhesive reflective tape ($1 mm^{2}$ of area) was glued at the centre of the back of each sample to increase the reflectivity of the laser beam and to avoid the reflected laser beam to be lost during surface displacement (Fig. 5(c));
• a dummy target represented by a piece of pink paper was glued on the sample holder to check the beam spot during the calibration phase prior proceeding with the samples irradiation (Fig. 5(d)).

An overview of the tested samples is given in Table 7.

**Table 7:** Materials investigated during the beam time.

Material sample	Sample Holder	Average thickness	Diameter
ASTM A213 T91 Steel (EN 1.4903 grade)	#1, upper housing	213.4 ± 6.2 μm	20 mm
Inconel 718	#1, lower housing	219.3 ± 4.9 μm	20 mm
EN AW 6082 T6 (Aluminium alloy)	#2, upper housing	405.4 ± 4.9 μm	20 mm
Titanium Grade 5 ELI (Grade 23)	#2, lower housing	219.9 ± 2.6 μm	20 mm

## 5 Irradiation experiments and testing protocol

### 5.1 The UNILAC M3-branch at GSI

**Figure 6:**
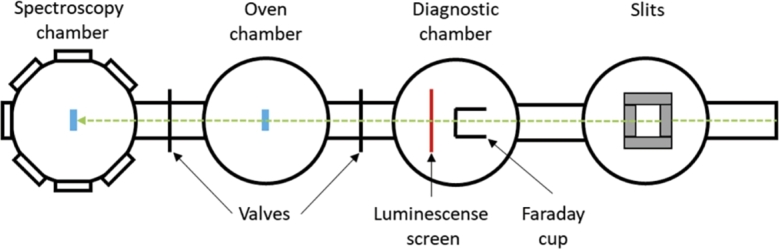
Schematic sketch of the M3 beamline of the GSI UNILAC accelerator. The beam is marked by a green dotted arrow, while the blue squares indicate the potential positions for irradiated samples [39].

The irradiation of the samples took place at the materials research branch of the GSI Helmholtz Center for Heavy Ion Research in Darmstadt (Germany) [56]. The uranium ion beams, coming from the linear accelerator UNILAC, have been passed through the M3-beamline to be directed towards the centre of the metallic specimens, mounted in sample holders. The impact between ions and target materials has occurred inside the spectroscopy chamber, outside of which the online measurement instruments have been placed. A schematic sketch of the M3-beamline is shown in Fig. 6.

### 5.2 In situ instrumentation

**Figure 7:**
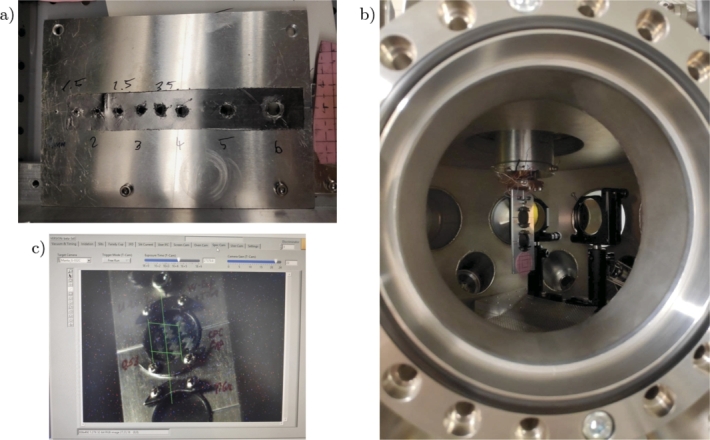
(a) Perforated aluminium plate with holes of different sizes: it is mounted inside the oven chamber to resize the square-shaped beam after passing through the slits, in order to obtain a smaller circular-shaped beam. (b) Interior view of the Spectroscopy chamber. (c) The software screen of the spectroscopy chamber camera focused on a sample before the irradiation test.

**Figure 8:**
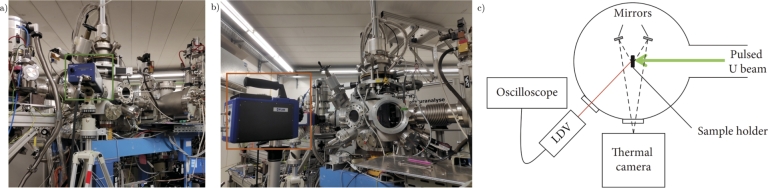
Arrangement of (a) Laser Doppler Vibrometer (green frame) and (b) thermal camera (orange frame) in front of the spectroscopy chamber of the M3-beamline. (c) A schematic representation of the top view configuration of online measurement instruments [39].

The surface velocity related to the beam-induced bending and the increase in temperature due to the deposition of the beam energy on the material samples were measured online by means of two instruments, respectively a Laser Doppler Vibrometer (LDV) and a thermal camera, whose technical specifications are reported in Table 8. The thermal camera was positioned orthogonally to the direction of the beam (Fig. 8(b)), and two infrared golden mirrors were mounted inside the spectroscopy chamber (as shown in Fig. 7(a) and (b)) to simultaneously reflect the two sides of the sample into the camera (Fig. 8(c)), thus making it possible to assess the temperature on both the front and back sides. The laser of the LDV was pointed through the free viewport of the chamber in the direction of the centre of the samples on the rear side, forming an angle of $45^{\circ }$ with respect to their surface (Fig. 8(a)).

**Table 8:** Specifics of the Laser Doppler Vibrometer and of the Thermal Camera adopted during the irradiation experiment.

Laser Doppler Vibrometer - High Speed System	

High Speed Sensor Head	Polytec OFV-525
Vibrometer Controller	Polytec OFV-5000-S
Max. acquisition velocity	20 m/s
Max. acquisition frequency	2.5 MHz
Measurement Range	10 - 2000 mm/s/V
Laser type	Helium neon, 633 nm, red laser beam

Thermal Camera	

Model	FLIR SC7000
Frame rate resolution	1475 frames per second
Spatial resolution	320 x 256 pixels
Thermal sensitivity	20 mK
Software	ALTAIR

### 5.3 Irradiation of the samples

Each sample holder was first clamped to a support in the top of the spectroscopy chamber so that it was kept stationary during the irradiation, as shown in Fig. 7(b). The procedure of opening and closing the chamber required the control of the vacuum system: before opening the chamber, which was maintained during the beam operation at 10^-7^ mbar (high vacuum condition), the vacuum valve was closed and the vent valve was opened to restore the room pressure in the chamber.

**Figure 9:**
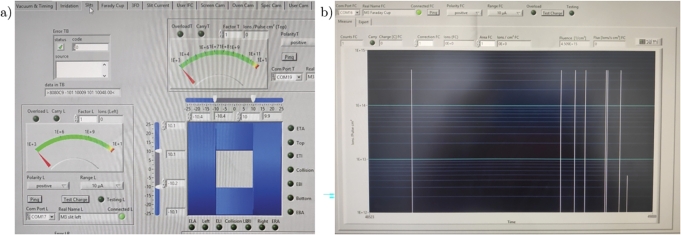
M3-beamline software screens during (a) calibration of the slits and (b) Faraday Cup in operation to verify the stability of the U-ion flux.

The calibration of the beam size and intensity followed. The shape of the U-ion beams was originally cut by four slits to obtain squared centred 20x20 mm^2^ beams before entering into the diagnostic chamber (Fig. 9(a)). Here, the beam was monitored and checked by the Faraday Cup and the luminescence screen, as shown in Fig. 9(b). The beam was therefore concentrated onto the 6 mm-diameter circular hole of the perforated collimating plate inserted in the oven chamber (Fig. 7(a)). This careful beam shaping procedure generates a super-Gaussian profile on the irradiated area, which can be effectively approximated to a flat-top profile without compromising accuracy. An ion beam with this profile optimises the uniformity of the irradiation and the dose accumulation over the short duration of the experimental test. However, it influences the thermal shock and dynamic stress conditions differently compared to the typical Gaussian profile of proton beams within accelerator facilities, making a direct correlation difficult.

The precise location of the circular beam spot (6 mm in diameter) at the centre of the samples was identified in terms of spatial coordinates and the electronic manipulator for driving the sample holders during the experiment was calibrated (see Fig. 7(c)).

The penetration depths of Uranium ions into the metallic targets were estimated through numerical simulations using the TRIM code, and the results have been included in Table 9. TRIM, a suite of programs designed to calculate the stopping power and range of ions in matter, employs a quantum mechanical approach to model ion-atom collisions. Specifically, TRIM-2013 was utilised for this study, which can determine the final distribution of ions within a complex target while assuming cylindrical symmetry in the ion distributions. The cylindrical axis, along which the projected range is measured, was assumed to be perpendicular to the target surface at the ion impact point. To ensure reliable statistical results with low uncertainty, the simulations were performed with 10000 incident U-ions for each target material — a number that has been proven in the literature to provide optimal estimates of ion-target interaction phenomena [48].

**Table 9:** U-ion beam parameters.

**Table:** Ion **Table:** Energy per nucleon **Table:** Ion energy **Table:** Pulse repetition rate **Table:** Pulse length **Table:** Beam intensity **Table:** Achieved Fluence **Table:** Irradiation time **Table:** Average current [MeV/u] [GeV] [Hz] [ μs] [ions cm^−2^/pulse] [ions cm^−2^] [s] [*μ*A] Uranium 4.8 1.14 1 100 5.0 x 10^9^ 2.0 x 10^13^ 4000 620
**Table:** T91 Steel Inconel 718 Al-6082-T6 Ti Grade 23 Penetration depth $d_{p}$ [ *μm*] 23.25 22.21 51.46 35.71

The target samples were finally exposed to 100 μs-long U-ion pulses with a beam repetition rate of 1 Hz and a kinetic energy of 4.8 MeV/u: this value of energy corresponds to the highest energy losses by ionising radiation (Bragg peak region) and allows fast accumulation of dose by heavy ions. For every minute of irradiation, 5x10^9^ uranium ions impact onto the target material. The calculation of the achieved fluence (strictly defined as the integral of particle flux over time [57]) at the end of a certain irradiation period may be significantly simplified under the assumption of constant beam repetition rate and intensity: the achieved fluence is thus equal to the product of the beam intensity and the time of exposition of the samples to the ion beam. The beam has not been run uninterruptedly towards the targets, but stopped at fixed time intervals and immediately restarted to allow the online measurement instruments to collect data: at each restart of the beam, the velocity signal generated by the first pulse and the temperature profile on both sides of the sample were recorded. The four samples were exposed to the U-ion beams for the same amount of time, till achieving a cumulative fluence of 2.0x10^13^ U-ions/cm^2^.

**Figure 10:**
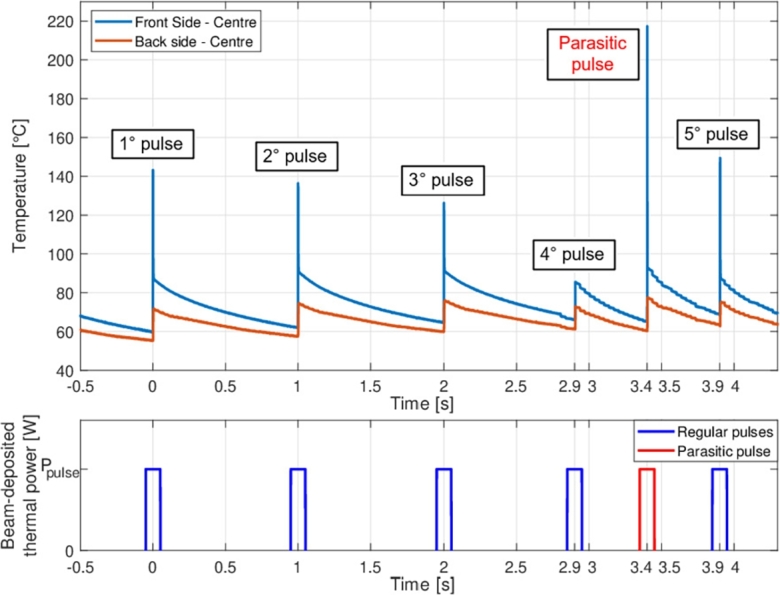
Time history of temperature and thermal power deposited by impacting pulses on the Titanium Grade 23 target at fluence 2x10^11^ U-ions cm^−2^. As can be easily observed, a parasitic pulse impacted the sample 0.5 seconds after the fourth regular pulse. The time length of the pulses is exaggerated for a simpler reading of the plot. The exact peak value is not always visible because the thermal camera captures images approximately every 670 μs. A higher sampling frequency would have been required to capture ion impacts accurately, as their pulse duration is only 100 μs. Therefore, the height of the thermal peaks does not necessarily indicate the maximum temperature reached immediately after the beam impact, but rather marks the moment of impact.

The proper progress of the experiment was also controlled by analysing the online data of the thermal camera. Temperature control was in fact paramount in preventing the components inside the spectroscopy chamber from overheating and in making sure that the steady-state temperatures of the metallic samples did not deviate too much from the room one. In this way, the annealing of the possible radiation-induced defects was impeded and the thermal properties of the target materials were assured to be the original ones. The thermal camera, furthermore, was used to verify the beam stability in terms of constant intensity and regular repetition rate of the ion pulses. The case of the temperature trend for the Ti-alloy sample exposed to the U-ion beam for five seconds (Fig. 10) is representative in this regard. Since the pulse repetition rate of the beam was set at a frequency of 1 Hz, a total of five peaks were expected to be recorded in five seconds of monitoring. In fact, an unexpected sixth pulse appears halfway between the fourth and fifth pulse: this parasitic pulse is attributable to the accidental transmission into the M3-beamline of a beam of U-ions destined for a different experiment that was being run at the same time. This parasitic pulse, with frequency of 0.2 Hz, was observed in the majority of TC curves: the cumulative fluence achieved during the irradiation test was higher by approximately 20% with respect to what was originally calculated. The thermal peaks recorded by the camera do not necessarily reflect the exact maximum temperature reached after each ion beam pulse, due to the camera's lower sampling frequency compared to the pulse duration. The key information provided by the thermal camera is the gradual temperature rise following each pulse, which confirms the deposition of energy by the ion beam. Although the height of the peaks may not always represent the maximum temperature, the subsequent temperature increase is sufficient to demonstrate the thermal impact of the irradiation. In cases where the peaks are captured accurately, the values offer additional insights for safety assessments and future thermo-mechanical analyses.

## 6 Results of the online measurements

### 6.1 Temperature profiles

The thermal effects induced by the quasi-instantaneous temperature increase induced by the impacting pulses in the region of the beam spot were analysed resorting to the thermal camera.

**Figure 11:**
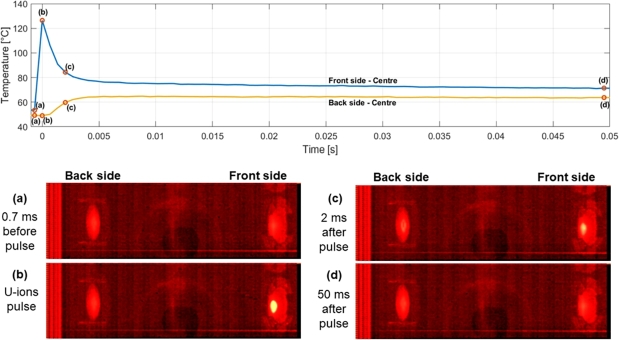
Detail of a 50 ms thermal profile of the fully irradiated Titanium Grade 23 sample. Four thermal images recorded by the TC are reported.

A 0.05-second extract of the thermal profile of the Titanium Grade 23 sample at the end of the irradiation period is represented in Fig. 11. The exact peak value was not always observable since the sampling frequency of the thermal camera (1475 Hz) allowed to acquire thermal images every about 670 μs, whilst the duration of the pulse was smaller (100 μs). The four images displayed in figure correspond to four main instants of temperature development:

(a) before the beam impact, when the temperature on the front and rear sides of the sample is almost uniform;
(b) during the beam impact, corresponding to the maximum temperature recorded on the front side;
(c) 2 ms after the impact, during the phase of rapid cooling of the impacted beam spot, when the heat spreads in the rear side and then in the whole sample;
(d) 50 ms after the impact, when the temperatures of the two sides are almost identical and the cooling is slow and constant.

**Figure 12:**
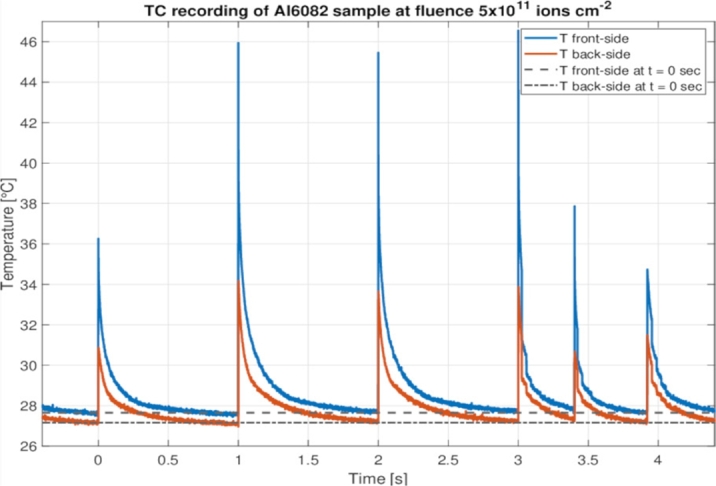
Five-second thermal profile of the Aluminium 6082 sample, captured by the thermal camera at a fluence level of 5x10^11^ ions cm^−2^. The graph illustrates that the quasi-steady-state temperature remains almost unaffected by the heat deposited on the target material by the U-ion beams.

**Figure 13:**
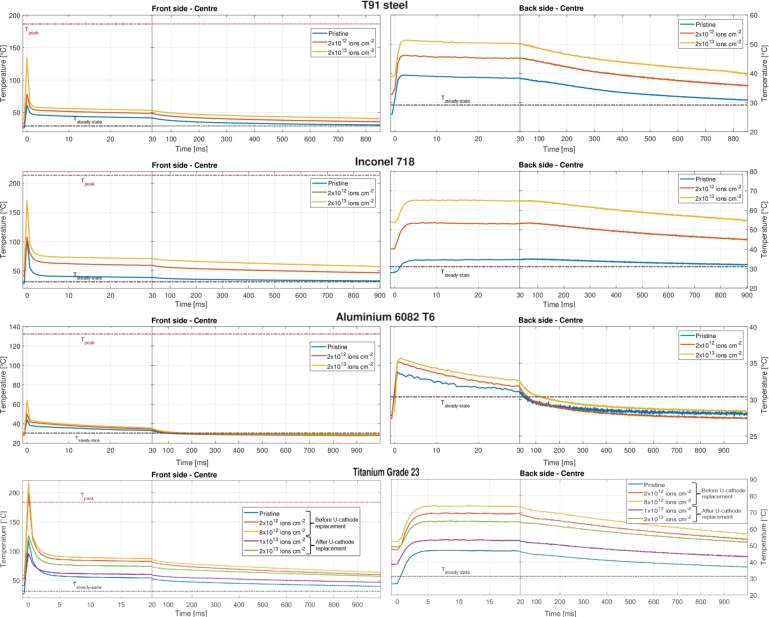
Comparison of the thermal profiles recorded by the thermal camera at different fluence levels, with peak and stationary temperatures (analytically computed and presented in Table 6), for samples in Steel T91 (thermal diffusion times: *τ*_R_≈ 13.2 s, *τ*_h_≈ 6 ms), Inconel 718 (thermal diffusion times: *τ*_R_≈ 31.4 s, *τ*_h_≈ 15.1 ms), Al-6082-T6 (thermal diffusion times: *τ*_R_≈ 1.35 s, *τ*_h_≈ 2.22 ms) and Titanium Grade 23 (thermal diffusion times: *τ*_R_≈ 34.8 s, *τ*_h_≈ 16.8 ms).
• The only significant variation between the thermal profiles of each material at different fluence levels is the initial temperature of the sample prior to the next uranium ions pulse, which is determined only by the amount of heat accumulated by previous pulses and not yet dissipated. Since a slowdown in the process of dissipation of the energy deposited in the beam spot (usually attributable to the detrimental effects of radiation damage on the values of the coefficients of specific heat and thermal expansion) did not seem to occur, it may be deduced that the latter coefficients remained constant and were not affected by the exposition to the U-ion beam.
• The thermal profiles of the Ti-alloy differ from those of other materials. The highest temperatures were not achieved for the highest levels of accumulated fluence, but rather for intermediate values. This was due to the replacement of the U cathode inside the accelerator at fluence 9x10^12^ ions cm^−2^, which interrupted the experiment for around 20 minutes and permitted the sample to almost totally cool down. When the experiment was finally restarted at intensity 4x10^9^ ions /(cm⋅2pulse) (lower than the value of 5x10^9^ maintained until then), the sample started warming up again, but the temperatures reached before beam interruption were not achieved again.
• A further aspect emerging from the analysis of the thermal curves is that the progressive increase in temperature with irradiation — mostly limited to a few tens of degrees — is extremely restricted in time and space. Accordingly, we do not expect that these thermal variations limited to tiny portions of metallic specimens can significantly affect their mechanical properties and dynamic behaviour.

The graphs in Fig. 13 show the temperature evolution of the four samples irradiated at different levels of fluence on both the front and rear sides. An analysis of these plots led to the following considerations:

• Materials having very high diffusivity, such as the Al-alloy, cool down quickly and are able to dissipate practically all of the heat received by the pulse before the following impact, with nearly identical thermal profiles at all the levels of fluence. The low quasi-steady-state temperatures maintained throughout the irradiation time suggest that the almost totality of deposited heat is dissipated through thermal conduction, initially toward the edges of the target sample, then along the sample holder and, finally, toward the containment structure of the spectroscopy chamber to which the sample holder is connected by a metallic support. The five-second thermal profile depicted in Fig. 12 illustrates the thermal stability of the Al-alloy sample in response to the impact of intense ion beams. The progressive heating of the Aluminium 6082 sample throughout the irradiation test, as the fluence increases, has been minimal, as is clearly shown in the figure. After six beam pulses in less than five seconds, the temperature recorded by the thermal camera (solid lines) and the initial temperature (dotted lines) continue to nearly coincide. This contrasts with the behaviour observed in Inconel and Titanium alloy samples, which exhibited more significant temperature rises over time. This limited heating further reinforces the stability of the Aluminium sample under high-fluence conditions.

### 6.2 Stress waves and vibrations

The analysis of the dynamic response of the target materials under irradiation of high-intensity short-pulsed U-ion beams is essential to evaluate the microstructural evolution as a result of the accumulation of radiation damage as well as to verify the onset of stress waves and other severe phenomena due to the effects of the beam impact on the metallic samples.

The velocity of the impacted samples, acquired with the laser doppler vibrometer, was examined to determine frequency and damping of the induced oscillations. In particular, the latter was estimated by evaluating the damping ratio *ζ* via logarithmic decrement method [58], while vibration and wave propagation frequencies were obtained increasing the frequency resolution of the FFT spectra by zero-padding the original time-domain signals [59]. The accuracy in determining the frequency peaks in the FFT spectra was assessed by the 3 dB bandwidth technique, commonly used in the signal processing field [60].

The results of the preliminary dynamic analyses, presented in Table 5, allowed an easier interpretation of the frequency peaks appearing in the FFT spectra of the acquired signals. Beam-induced stress waves were not detected: as anticipated, primary waves, characterised by propagation frequencies of the order of tens of MHz (see Table 5), were out of the range of measure of the vibrometer. Shear vertical waves, which are particularly difficult to identify because of their dispersed nature, especially in the presence of low-intensity excitation sources [53] were not detected as well. The frequency analysis was thus reduced to the identification of the experimental natural frequency of vibration and, in particular, to the research of the value of the first bending frequency ($f_{b0}$) at different levels of accumulated fluence.

**Figure 14:**
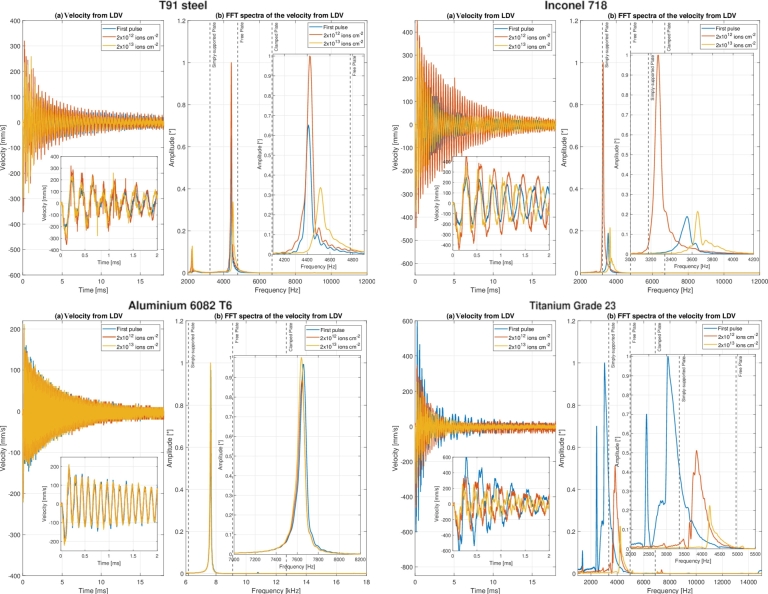
(a) Time-dependent velocity signal measured by LDV and (b) corresponding FFT spectra at the rear side of disc-shaped samples in Steel T91, Inconel 718, Aluminium 6082 and Titanium Grade 23 for the irradiation with 1.14 GeV Uranium ions at different levels of fluence.

The plots in Fig. 14 represent the time-dependent velocity signal (shown in Fig. 14(a), with a zoomed view on the first 2 ms of the signal) and the corresponding FFT spectra (shown in Fig. 14(b)) recorded by the vibrometer at the rear side of each disc-shaped sample under U-ion irradiation at different levels of fluence. The plots in the frequency domain have been normalised with respect to the highest peak value (which is supposedly the peak corresponding to the first bending frequency) to facilitate its readability. Significant considerations arising from these plots are reported separately in the following for each metallic sample. Regardless the dynamic analyses of the single materials, it can be definitively stated that the frequency shifts of that magnitude cannot be ascribed to alterations in the mechanical properties of limited irradiated portions of the samples due to an increase in temperature, as previously discussed in Section 6.1.

#### 6.2.1 Target sample in steel T91

In the pristine sample, a harmonic oscillation with frequency 4.441 kHz is generated: this frequency can be ascribed to first bending frequency and its value is intermediate between the values of frequency analytically found for simply-supported (3.234 kHz) and free sample (4.778 kHz) boundary conditions (see Table 5). The damping is equal to 0.0229, with the amplitude that decreases from the initial velocity of 200 mm s^−1^ to 20 mm s^−1^ in less than 20 milliseconds in the case of the first beam-induced impact on the target sample. Continuing with the irradiation, the peak frequency increases up to 4.517 kHz at the cumulative fluence of 2x10^13^ U-ions cm^−2^. The 100 Hz increase in frequency could be caused by both the change in boundary conditions (due to the progressive rise in temperature of the sample with relative expansion/contraction at the boundary) and by the hardening of the material (and the corresponding increase of Young's modulus in the irradiated area). Besides, since the steady-state temperature of the steel sample increases only slightly during irradiation, it can be excluded that the shift in frequency may be caused by temperature-induced changes in the physical properties of the metallic sample.

**Figure 15:**
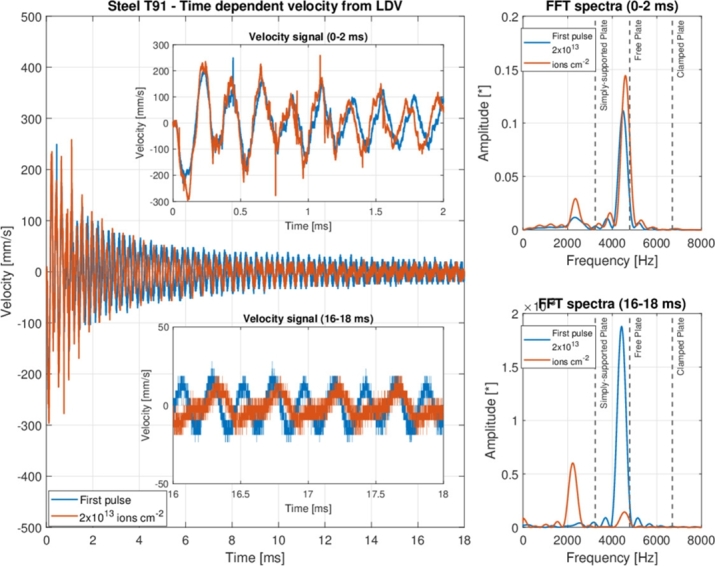
Steel T91 sample — On the left, the time-dependent velocity signal measured by the LDV, with two zoom-ins related to the first 2 ms (0-2 ms) and last 2 ms (16-18 ms) and, on the right, the FFT spectra corresponding to the two parts of the signal of which the magnification was carried out.

The peak observable at around 2000 Hz requires further examination. To this end, a more in-depth analysis of the first 2 ms (from 0 to 2 ms) and of the last 2 ms (from 16 ms to 18 ms) of the velocity signal related to the first and last pulse was made (see Fig. 15). The zoom-ins related to these signal parts are presented in figure in the time (on the left) and frequency domains (on the right). In the first 2 ms, the frequency spectra are similar to the ones already discussed for the entire signal, with the maximum peak (corresponding to the first bending frequency) that slightly shifts forward. On the contrary, a different behaviour can be observed in the last 2 ms: while the frequency spectra for the first-pulse signal is essentially identical to the one found for the first 2 ms, the frequency spectra associated with the last pulse (fully-accumulated fluence) show the maximum value at a frequency that is about half that of the first bending frequency. A peak at this frequency of vibration is still visible, but its amplitude is significantly lower than the one at around 2200 Hz. Such a behaviour could be ascribed to the change of the boundary conditions that the sample experienced while the velocity signal was being damped: with the decreasing amplitude of the velocity signal, the peak frequency progressively shifted from the free condition towards the simply-supported one as a result of the decrement of the amplitude of the oscillations fostering an enhanced adherence to the sample holder. This peculiar aspect seems to have occurred only for the fully-irradiated sample, namely when the axial and radial clamping effects between the outer radius of the steel sample and its sample holder were reinforced as a result of the beam-induced heating.

#### 6.2.2 Target sample in Inconel 718

The amplitude of the velocity signal and the damping (ζ=0.0282) is quite comparable to those observed for the steel sample since their values of Young's modulus and coefficient of thermal expansion do not differ significantly. The only peak in the frequency domain observed in Fig. 14 (at 3.550 kHz) is related to the first bending frequency. Even in this case, the first mode of vibration detected by the vibrometer lies between the analytically computed values for simply supported (3.175 kHz) and free (4.695 kHz) conditions. At the beginning of the irradiation, the amplitude of the velocity signal increases with fluence and the frequency $f_{b0}$ gradually decreases up to ∼ 3.272 kHz at fluence 2x10^12^ ions cm^−2^. Then, from this point on, the frequency starts to almost linearly increase until it settles at 3.656 kHz at the maximum accumulated fluence. It is not immediately clear why the frequency shifts as the accumulated fluence rises: this complex behaviour is thought to be due to the change of the boundary conditions as the temperature increases as well as the change of mechanical properties after irradiation.

#### 6.2.3 Target sample in Aluminium 6082

Compared to the above two cases, the signal attenuation is less pronounced (damping ratio equal to 0.0136) and the velocity signal has lower values in amplitude (maximum values below the 200 mm s^−1^, see Fig. 14). In the quasi-pristine sample, the harmonic oscillation with frequency 7.656 kHz (in the middle between the analytical values of 6.174 kHz for simply-supported condition and 9.108 kHz for free condition) is definitely attributable to the first bending frequency. With increasing fluence, the frequency only slightly fluctuates around the value of 7.6 kHz. These findings suggest that it is unlikely that significant changes in material properties occurred within the area irradiated by the ion beam.

#### 6.2.4 Target sample in Titanium Grade 23

The signal attenuation is similar to what was found for the Inconel sample, having equal thickness, with a damping ratio of 0.0280 for the Ti-alloy sample. The amplitude of the velocity signal is significantly reduced with increasing the accumulated fluence: this drop is partly attributable to the decrease of the intensity of the ion beam after the replacement of the Uranium cathode at fluence $9\times 10^{12}$ ions cm^−2^ (see Fig. 14). The value of the first bending frequency rises considerably with increasing U-ion fluence, from 3.39 kHz for the quasi-pristine sample at the first impact to 4.217 kHz at the maximum achieved fluence. The comparison with the values analytically found reveals that the boundary condition of free disc is absolutely appropriate for describing the natural vibration frequency of the titanium alloy sample (3.361 kHz from analytical calculations). The monotonous and growing trend of the frequency could be ascribed to two phenomena, such as the microscopic defect accumulation induced by U-ions radiation, with consequent material hardening and increase of the Young's modulus within the beam spot, and the reinforcement of the tightening condition at the edges of the sample, due to the increase in temperature and the corresponding expansion of the sample. Unlike what was previously seen for the signals of the other materials, a large number of peaks is observable in the frequency domain. Two peaks which can not be associated with any bending modes were detected: one fluence-independent peak at 1.36 kHz and another peak, whose frequency rises with increasing fluence, that precedes the peak corresponding to first bending frequency by a few hundred Hz.

**Figure 16:**
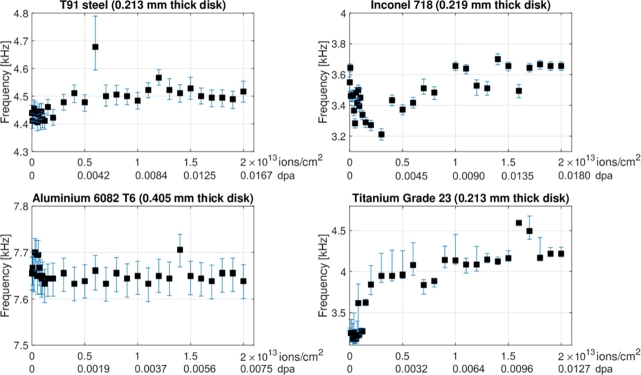
Measured bending frequency of the four disc-shaped samples with diameter 2 cm as a function of accumulated 4.8 MeV/u Uranium ion fluence (ions cm^−2^) and corresponding irradiation damage (dpa), as calculated with the SRIM-2013 code (Quick-calculation option) [47].

Fig. 16 shows how the first bending frequency, measured by LDV for the four samples subjected to irradiation during the experiment, changed as a function of accumulated U-ion fluence and corresponding radiation damage (dpa), as computed by SRIM-2013 software. The confidence intervals of the first bending frequencies were represented by vertical error bars. The lower and upper values of these bars were calculated using the 3 dB bandwidth technique previously described. By presenting both ion fluence and dpa on the x-axes, the plots enable a clear and direct understanding of the relationship between the ion fluence to which the metallic samples were exposed and the resulting damage levels. The inclusion of these latter data, to whose analysis adequate space will be allocated in a forthcoming study concerning thermomechanical simulations and particle transport calculations, provides readers with a more direct framework for evaluating the irradiation conditions of the experiment. Finally, a summary of the frequency measured at four different levels of accumulated fluence for the investigated materials is reported in Table 10.

**Table 10:** Bending frequency of the four material samples at certain levels of fluence.

Material sample	**Table:** Measured bending frequency [kHz] at fluence [U-ions cm^−2^]			
	First pulse	2x10^11^	2x10^12^	2x10^13^
ASTM A213 T91 Steel	4.411	4.456	4.478	4.517
Inconel 718	3.550	3.461	3.272	3.656
EN AW 6082 T6 (Aluminium alloy)	7.656	7.656	7.644	7.639
Titanium Grade 5 ELI (Grade 23)	3.039	3.250	3.844	4.217

## 7 Post-irradiation examinations: results and discussion

A series of test were performed on the irradiated and pristine samples to estimate the effects of ion exposition on the hardness and Young's modulus of the analysed materials.

### 7.1 Microindentation tests

**Figure 17:**
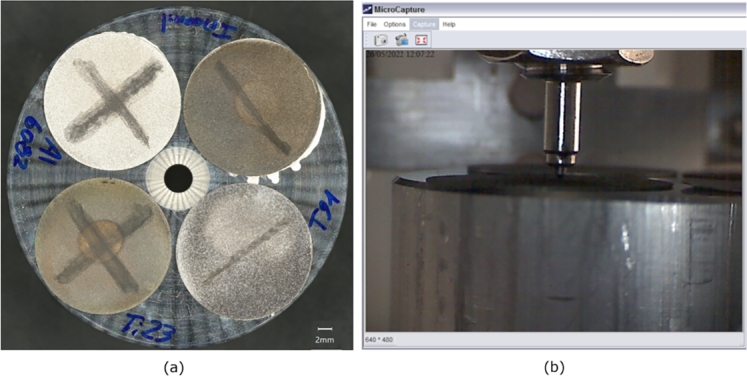
(a) Picture of the aluminium support (with a diameter of 5 cm and thickness of 1.5 cm) for microindentation test on the surface of which the four metallic samples exposed to U-ion beams were glued. (b) Capture from the NanoTest software camera of the indenter and of the Ti-alloy sample, extremely useful to align the indenter in correspondence of the sample irradiated area.

The microindentation test, held at the laboratory of the Materials Research Department at GSI, was performed by employing a NanoTest Vantage Indenter produced by Micro Materials [61], [62]. The metallic samples, once extracted from the holders, were made to adhere to an aluminium support (shown in Fig. 17(a)) using cyanoacrylate glue, making sure that the glue layer was as thin as possible to prevent compromising the microindentation test. A second aluminium support with pristine samples was prepared in the same way. These supports were then placed in the sample stub of the testing machine and securely fixed by a screw at the centre of the support. A Berkovich tip (i.e. a diamond indenter tip of three-sided pyramidal shape with a very flat profile) was adopted for the tests in order to ensure accurate measurements at shallow depths. The interaction between the indenter and the irradiated Ti-alloy specimen is presented in the picture in Fig. 17(b), taken by the integrated optical microscope utilised for inspecting the sample locations during the test. A maximum penetration depth of 2000 nm was selected for all the measurements. This depth, equal to one-tenth of the U-ions penetration depth inside the steel sample, is small enough to minimise the influence of the non-irradiated substrate below the irradiated layer and, at the same time, to remain in the region of constant electronic energy loss. Literature shows that the stopping power remains nearly constant at shallow depths due to the dominance of electronic interactions [63]. Given that the electronic stopping power remains nearly constant at these depths, the dependency between the damage profile and penetration depth of the indents can be considered negligible without compromising the accuracy of the microindentation results. However, we recognise that the selected depth makes the measurements particularly sensitive to the location and orientation of the few penetrable metal grains present at such a shallow depth. For each sample, twenty-five microindentation measurements were performed, in a square 5x5 pattern. The indentation load-displacement data recorded during the cycles of loading and unloading were analysed by the Oliver-Pharr method to determine the elastic modulus of each sample [64], [65]. The accuracy of modulus measurement depends on multiple factors which were adjusted and evaluated during the calibration stage, such as the maximum load, the holding and release times of the indenter and the diamond area function.

**Figure 18:**
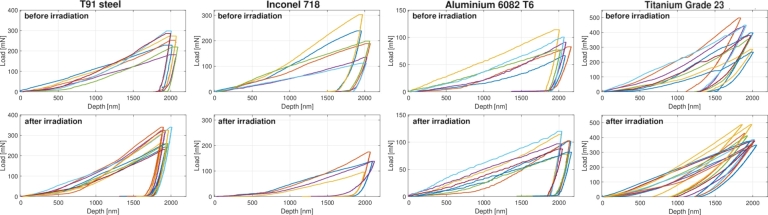
Selected Load-Displacement curves from microindentation analysis of pristine and irradiated (at the maximum accumulated fluence of 2*x*10^13^ ions cm^−2^) samples in steel T91, Inconel 718, Aluminium 6082 T6 and Titanium Grade 23.

Fig. 18 presents the load-displacement curves relating to each pristine and ion-irradiated metallic sample which were taken into account for measuring the Young's modulus. The difficulty of performing the test resulted into the acquisition of a certain number of microindentation curves lacking the necessary quality. This explains why the number of curves is not the same for each graph.

While these tests provide valuable insights, we advise caution in interpreting the results due to the inherent sensitivity of microindentation at shallow depths, as reflected by the high standard deviations observed in the measurement data. These results should be viewed as indicative of trends rather than definitive conclusions regarding Young's modulus variations after irradiation.

In Table 11, the results of the microindentation tests in terms of Young's modulus *E* for all the samples investigated are reported. The results on the pristine specimens show excellent agreement with the values coming from the data sheets, supporting the validity of the indentation procedure for characterising these materials. The pristine specimens show excellent agreement with the data sheet values, supporting the validity of the indentation procedure for characterising these materials. Furthermore, the similarity between the irradiated and pristine values for the Al-alloy, steel T91, and Inconel 718 samples suggests that no significant phase changes occurred due to ion irradiation, as expected given the low displacements per atom (dpa) accumulated. However, the increase in Young's modulus of approximately 54 GPa in the central beam area of the irradiated Titanium Grade 23 sample, though significant, requires further validation. These findings suggest potential irradiation-induced modifications, but we emphasise that the microindentation results should be regarded as informative rather than conclusive, pending future validation through complementary techniques.

**Table 11:** Results of the microindentation analysis (average values and standard variations) for pristine and irradiated samples in terms of Young's modulus.

Material sample	Young's modulus [GPa]		
	Data sheets	Microindentation	
	Pristine	Pristine	After irradiation
ASTM A213 T91 Steel	207	199 ± 47	222 ± 45
Inconel 718	203	194 ± 57	207 ± 45
EN AW 6082 T6 (Aluminium alloy)	70	77 ± 27	68 ± 17
Titanium Grade 5 ELI (Grade 23)	113.8	115 ± 25	169 ± 9

### 7.2 SEM analyses

SEM analyses were carried out at CERN, in the laboratory of the Materials, Metrology and Non-Destructive Testing Section. The Sigma-500 scanning electron microscope system from ZEISS was utilised for the microscopic investigation of the irradiated samples [66].

**Figure 19:**
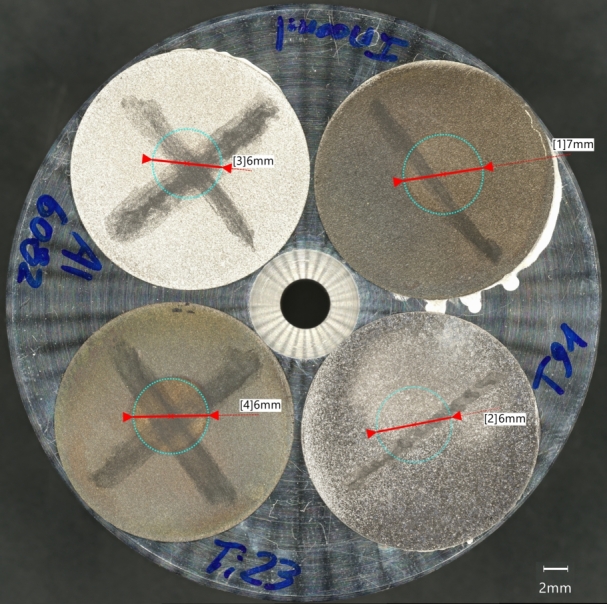
High-quality picture of the aluminium sample support with the irradiated metallic specimens, taken by the Sigma-500 SEM system. The circles mark the irradiation area, of which the diameter is indicated.

A high-quality picture of the four target specimens glued to the metallic support used for the microindentation test was taken by using the Sigma-500 SEM system (Fig. 19). Surface observations were taken at the irradiated areas (marked with the circles) and next to the edge of the samples, where the material is still in pristine conditions. The samples in Ti-alloy and Inconel 718 show a slight visual discolouration in the irradiation-affected area. The reason for the colour change is not established, but it is most likely related to oxide layer thickness variations.

**Figure 20:**
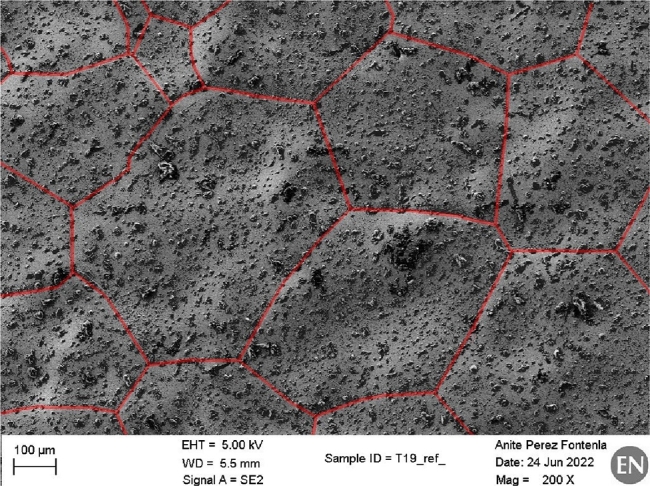
SEM micrograph with 200X magnification of the pristine area of the Steel T91 sample. The red lines mark the grain boundaries.

**Figure 21:**
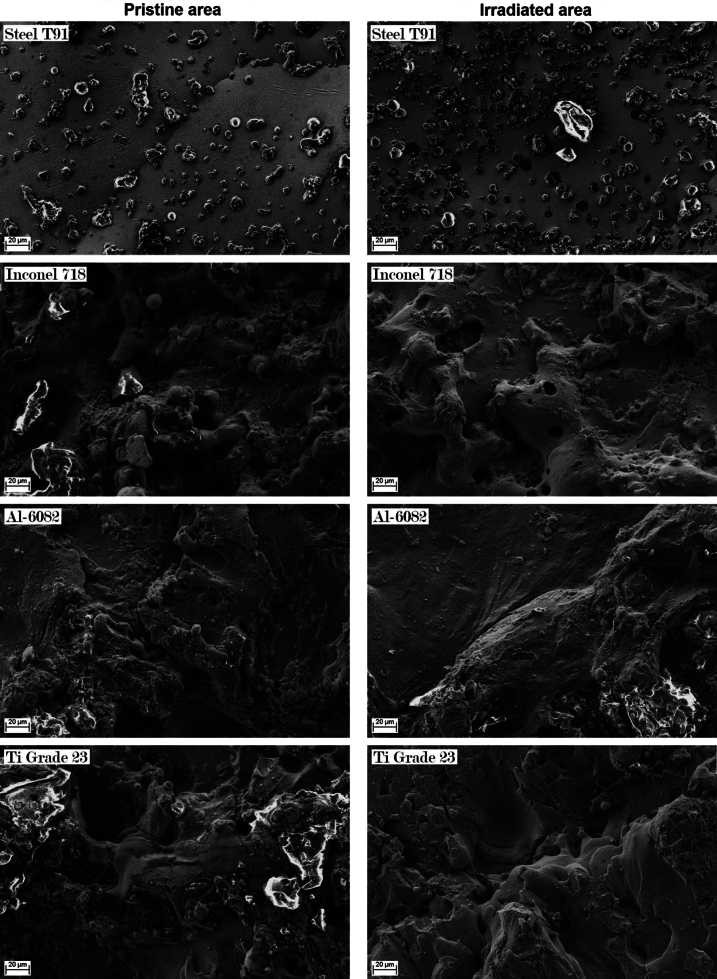
SEM micrographs with 1000X magnification of both pristine and irradiated areas of the four metallic specimens tested.

Fig. 21 presents SEM observations of the pristine (on the left) and irradiated (on the right) areas of the four metallic samples. The quality of these observations is closely related to the type of surface treatments carried out after sample production: oxides were produced on the sample surfaces by the wire electrical discharge machining (EDM) technique used to cut metallic samples from round bars. The mechanical polishing on the surfaces did not remove completely these oxides: the SEM observations show the typical EDM microstructure, with molten droplet structures of different sizes, for all the materials, with the exception of the steel T91. Micrographs of T91 sample present smoother and less rough surfaces than the ones of the other three materials: molten droplet structures are absent and grain shape and orientation are immediately recognisable, as shown in Fig. 20, where grain boundaries are marked by red lines.

There are no relevant differences between the observations of pristine and irradiated surfaces. The particles visible on all the surfaces are dust from manipulation and are not related in any way to specific traits of the materials under investigation nor to changes due to the ion irradiation. Since the sample surfaces were not prepared for microstructural examination with proper polishing and etching treatments, information about the irradiation effects on the grain size is unavailable.

## 8 Conclusion

Short-pulsed uranium ion beam exposure with 1.14 GeV kinetic energy was conducted at the materials research branch of the linear accelerator UNILAC at Darmstadt GSI on four target samples, made of metals deemed of major interest for beam-window applications in high-power accelerator facilities. All metallic targets were exposed to the ion beams until achieving a cumulative fluence of 2.0 x 10^13^ U-ions/cm^2^. During the irradiation experiments, the beam was regularly interrupted at fixed time intervals and, soon after, restarted to enable data collection by online measurement instruments. The beam-induced heating of the samples was controlled with a thermal camera, while the dynamic response of the targets was monitored by recording the surface velocity signal of the samples using a Laser Doppler Vibrometer. Microindentation tests and SEM observations were performed after irradiation to validate the results of in-situ measurements and to identify possible microstructural changes due to ion beam exposition. The outcomes of these experimental investigations can be summarised as follows:

**Steel T91**: The stationary temperature was observed to only slightly rise (less than 10 ^∘^C) between the beginning and end of the irradiation period, highlighting the good thermal properties of such steel grade (which did not seem to be affected by ion exposition) under intense pulsed beams. Concerning the dynamic analysis, the observed 100 Hz increase in first bending frequency from pristine to fully-irradiated sample could probably be ascribed to a change in boundary conditions due to the — albeit limited — heating of the sample holder assembly. Radiation hardening of the metal seems to be ruled out from the possible causes of the frequency shift, as proved by the microindentation tests (Young's modulus basically unvaried) and by the SEM observations (grain size did not change, as shown in Fig. 20). Besides, the effective influence of the beam-induced heating on the clamping effects between the steel sample and its holder was proven by the in-depth analysis of the peak observable at around 2000 Hz (Fig. 15).
**Inconel 718**: The poor thermal qualities of this Nickel superalloy were observed to prevent the heat deposited by the ion beams from completely spreading outside the sample before the next pulse, thus causing a constant increase of the steady-state temperature (around 54 ^∘^C before the last pulse) and higher peak temperatures. The results of the dynamic response did not allow an easy interpretation as the first bending frequency was observed not to follow a monotonous trend with increasing accumulated fluence. The amount of increase in Young's modulus (about 10 MPa) measured by microindentation was not sufficient to explain the steep growth in bending frequency noticed between $3\times 10^{12}$ and $10\times 10^{12}$ U-ions/cm^2^. A possible explanation sees the first descending phase as being due to the initial adjustment of the sample inside the ring-shaped rim, whilst the subsequent rise in frequency may be ascribable to the increase of the tightening effect on the boundary on account of the gradual increase of stationary temperature during irradiation.
**Aluminum 6082**: The excellent thermal properties of the aluminium alloy ensured complete diffusion of the heat deposited by a beam pulse prior to the subsequent one, keeping the stationary temperature constant. Considering that the first bending frequency was observed to only slightly fluctuate about the value of 7.6 kHz with increasing accumulated fluence, it could be asserted that the dynamic analyses did not highlight any beam-induced changes in the elastic modulus of the material, as demonstrated by the negligible reduction in E measured by microindentation testing.
**Ti Gr23**: The low thermal diffusivity of the titanium alloy affected the heat spreading outside the beam spot, resulting in high peak temperatures (above 200 ^∘^C) and increasingly higher stationary temperatures during the irradiation test. The velocity signals recorded by the vibrometer were reduced significantly in amplitude with increasing accumulated fluence and the FFT spectra showed many peaks not attributable to any vibration modes. The significant change in the first bending frequency content (around 1.2 kHz) could be influenced by multiple factors, such as the radiation-induced material hardening, which, however, cannot be confirmed with certainty due to the limitations of the microindentation test. Although the microindentation results suggest a 55 GPa increase in Young's modulus, this finding should be interpreted with caution, and further validation is required to definitively attribute this change to irradiation effects. The tightening condition on the sample boundaries resulting from thermal expansion likely also played a role in this frequency shift. Given these uncertainties, no definitive conclusions can be drawn regarding radiation-induced hardening based solely on these results. No indication about the radiation-induced hardening could be provided from SEM observations.

Further experiments will be required to test these materials in close-to-operative conditions (e.g. under intense pulsed proton beam irradiation, in presence of pressure difference and in the highly-corrosive environments of the future accelerator-driven systems). Particle transport simulations will need to be performed to accurately assess the radiation dose absorbed by the metallic samples and refine the precision of dpa calculations. Moreover, these simulations will be crucial in laying the groundwork for the upcoming thermomechanical simulations, whose primary aim is to investigate more deeply the effects associated with the temperature-induced variations at the sample boundaries, allowing to obtain additional insight into the physics of the observed thermo-mechanical responses of the materials. Moreover, given their independence on changes in the boundary conditions of the samples - which proved to be difficult to control in the experimental tests - the implementation of an online strategy to monitor the samples dynamics based on the acquisition of the beam-induced propagation phenomena rather than on their vibrations is advised.

## CRediT authorship contribution statement

**Lorenzo Notari:** Writing – review & editing, Writing – original draft, Visualization, Validation, Software, Resources, Methodology, Investigation, Formal analysis, Data curation, Conceptualization. **Michele Pasquali:** Writing – review & editing, Validation, Supervision, Project administration, Methodology, Formal analysis, Data curation, Conceptualization. **Federico Carra:** Writing – review & editing, Validation, Supervision, Project administration. **Marcello Losasso:** Writing – review & editing, Project administration, Funding acquisition. **Jorge Guardia-Valenzuela:** Formal analysis, Data curation. **Marilena Tomut:** Methodology, Funding acquisition, Conceptualization.

## Declaration of Competing Interest

The authors declare that they have no known competing financial interests or personal relationships that could have appeared to influence the work reported in this paper.

## Data Availability

Data will be made available on request.
